# Model for Calculating Impact Force for Individualized Hip Fracture Prediction During a Fall

**DOI:** 10.1155/aort/9541321

**Published:** 2025-12-05

**Authors:** Alisha Agarwal, Daniel Kargilis, Nishtha Gupta, Michael Chang, Rui Feng, Gregory Chang, Chamith S. Rajapakse

**Affiliations:** ^1^ Department of Medicine, Sidney Kimmel Medical College, Philadelphia, Pennsylvania, USA, jefferson.edu; ^2^ Department of Medicine, University of Pennsylvania, Philadelphia, Pennsylvania, USA, upenn.edu; ^3^ Department of Medicine, John Hopkins University School of Medicine, Baltimore, Maryland, USA, jhu.edu; ^4^ Department of Medicine, Drexel University College of Medicine, Philadelphia, Pennsylvania, USA, drexel.edu; ^5^ Department of Medicine, New York University, New York, New York, USA, nyu.edu

**Keywords:** bone strength, fracture risk, hip fracture prediction models, MRI-based finite-element analysis, osteoporosis, patient-specific characteristics

## Abstract

Osteoporotic‐related weakening of bone is a common cause of hip fractures. The standard of care for the diagnosis and management of osteoporosis is the dual‐energy x‐ray absorptiometry bone mineral density T‐scores. Many individuals considered nonosteoporotic, however, still sustain fractures since these tools do not incorporate vital bone parameters and subject‐specific characteristics. The purpose of this work was to (1) develop a simple analytical model for estimating the force exerted on the femur during a fall (i.e., impact force) based on measurable patient metrics and (2) define a quantifiable fracture risk index by comparing finite‐element‐derived bone strength and impact force, which could be validated in a cohort of human subjects. Aggregated regression models were derived for estimating impact force based on patient age, weight, height, and soft tissue thickness. Patients with a history of hip fractures were then compared to a matched nonfracture group via the bone strength index (BSI), defined as the ratio between bone strength and maximum impact force. The BSI was lower in the fracture group compared to the control group by 0.23 (*p* = 0.045). The combination of patient‐specific impact force on the femur during a fall and bone strength could provide additional insights into osteoporotic hip fracture risk alongside standard risk assessments.

## 1. Introduction

Each year, an estimated 1.5 million individuals suffer a fracture due to bone disease with the most common underlying pathology being osteoporosis, a debilitating bone disease characterized by low bone mass and resulting skeletal fragility [1]. Annual healthcare spending due to osteoporotic fractures amounts to $20 billion dollars, a majority of which are related to fractures in elderly patients [2]. One of the most catastrophic of all fractures sustained in elderly populations is a hip fracture due to falling from a standing height. These injuries have been associated with serious consequences such as significant comorbidities, loss of independence, and mortality with up to 20% of patients dying within a year of fracture [3]. As life expectancy continues to increase and a greater proportion of the total population is comprised of elderly individuals, the prevalence of hip fractures and their subsequent costs are expected to continue to rise [3].

One way to facilitate the diagnosis and management of osteoporosis is using bone fracture risk calculators. There are numerous osteoporotic risk prediction tools available, the most widely accepted being fracture risk assessment tool (FRAX) scores, which utilize important measurements such as the dual‐energy x‐ray absorptiometry bone mineral density (DXA BMD). Risk calculators can be used to determine whether prophylactic osteoporotic fracture treatment should be utilized or not. Despite the wide popularity of these tools, many individuals still sustain fractures who are classified as nonosteoporotic by current standards. DXA is limited by its lack of incorporation of trabecular microstructure, bone composition, and porosity, all important elements of femoral strength [4]. The only bone‐related factor that FRAX directly accounts for is the bone mineral density.

Finite‐element analysis applied to three‐dimensional models of the femur derived from computed tomography or magnetic resonance imaging (MRI) has shown promise for an improved assessment of bone strength as it takes into account the bone’s three‐dimensional architecture in addition to bone density [5–7]. Previous work has shown that proximal femur strength calculated from high‐resolution MR images using finite‐element analysis is highly correlated with experimentally obtained gold standard values via mechanical the testing of cadaveric human femora [8]. It is important to note that an individual will break the femur during a fall if the impact force felt by the femur is greater than the bone strength. Therefore, knowing the impact force in addition to bone strength is important for predicting hip fracture risk for an individual [9, 10]. While femoral strength can be computed with high accuracy from image‐based finite‐element models, impact force exerted on the femur during a fall has to be estimated using an empirical equation that takes as input an individual’s weight, height, and image (e.g., whole‐body DXA)‐derived trochanteric soft tissue thickness (STT) as shown in Ref. [9]. Furthermore, Bouxsein et al. found that differences in the trochanteric STT between patients with incident hip fractures compared to controls were associated with differences in the ability of the femur to attenuate forces during a fall and, in turn, different fracture outcomes [9].

The goals of this study were to (1) develop multiple regression models that could readily estimate impact force on the femur during a sideways fall based on different subject‐specific characteristics including age, weight, height, and STT and to (2) develop a metric defined as the bone strength index (BSI) for assessing patient‐specific hip fracture risk using the ratio between MRI‐derived bone strength and impact force, and validate this approach on hip fracture subjects compared to matched controls.

## 2. Methods

### 2.1. Data Collection

A literature search was conducted to identify studies that describe factors that affect the impact force at the proximal femur during a sideways fall impacting the hip. Key phrases such as “osteoporotic hip fracture prediction” were entered into the search box of the PubMed database. Age, weight, height, and STT between the greater trochanter and the skin were determined to be major factors influencing the impact force (*n* = 237 subjects, 11 total studies). For each of the studies identified, a univariate equation was recorded describing the relationship between impact force as the dependent variable and individual parameters as the independent variable. When sources did not explicitly mention a definitive equation, an estimated model was derived by generating a best fit line to minimize the least‐square error based on data points shown on correlation plots or tables within the literature.

### 2.2. Aggregate Regression Model Derivation

For each factor found to influence impact force at the hip (i.e., age, weight, height, and STT), a linear equation was derived to describe the impact force as the dependent variable by combining data from multiple studies. Literature data for each parameter were aggregated to generate a best fit line between the independent parameter and the dependent impact force. Slope and intercept of each equation were derived by minimizing the least‐square error.

### 2.3. Calculating BSI

BSI, a quantitative proxy for hip fracture risk, is defined as the ratio of the patient’s bone strength (calculated using finite‐element analysis, as described below) to the maximum impact force (derived from the regression models, as described above). A proximal femur was predicted to fracture during a sideways fall if the BSI is less than unity.

### 2.4. Analysis of Patient Data

To test the clinical utility of using the BSI for predicting fracture risk, a retrospective study was conducted comparing 10 patients who had sustained an osteoporotic hip fracture against ten nonhip fracture controls with reported osteoporosis or osteopenia that were matched based on age (difference ±0.3 years), height (difference ±0.04 m), weight (difference ±6 kg) and BMI (difference ±1 kg/m^2^) (Table 1). Patient characteristics were recorded at the time of MRI. STT between the greater trochanter and skin was measured using magnetic resonance images of the hip. Informed consent was obtained from all participants included in the study.

**Table 1:** Patient characteristics.

	Control *N* = 10	Hip fracture *N* = 10	*p* value
Age (years)	64.0 [54.0–68.8]	63.5 [53.5–68.8]	0.909
Height (*m*)	1.60 [1.54–1.65]	1.63 [1.58–1.68]	0.363
Weight (kg)	52.4 [47.0–60.1]	56.2 [50.2–65.8]	0.273
BMI (kg/m^2^)	19.9 [19.4–24.2]	21.5 [19.6–24.2]	0.650
MRI side	Right (*n* = 3) Left (*n* = 7)	Right (*n* = 8) Left (*n* = 2)	—
Gender	2 males 8 females	2 males 8 females	—
Number of current smokers	0	1	—
Glucocorticoid therapy use	0	1	—
Previous nonhip fracture	6	10	—
Average number of previous fractures	1.0	2.2	—

This study included postmenopausal women with prevalent fragility fracture of the femoral neck and healthy controls matched for age, race, and BMI from the ages 50–90 years. Postmenopausal status is defined as the lack of menstruation for 1 year. Fractures were confirmed by a radiologist and defined as a low‐trauma fracture due to a fall from a standing height or less. The exclusion criteria included contraindications to MRI (e.g., cardiac pacemaker) and fractures resulting from high trauma (e.g., motor vehicle collision) or due to a pathologic bone lesion.

### 2.5. MRI

For all participants, the nondominant and nonfractured hip was imaged with a 3‐T whole‐body MR imager (Siemens, Erlangen, Germany) using a 26‐element body matrix coil wrapped and secured around the hip anteriorly and eight elements from a spine coil posteriorly. Participants were scanned using a three‐dimensional fast low‐angle shot sequence (FLASH) with the following parameters: echo time (TE)/repetition time (TR) of 4.92 ms/37 ms, voxel size of 0.234 mm × 0.234 mm × 1.5 mm, 60 coronal slices, an acceleration (generalized autocalibrating partially parallel acquisition) factor of 2, and an acquisition time of 15 min 18 s.

### 2.6. Femur Segmentation

Extraction of the proximal femur from the background in hip MR images was performed using the in‐house‐developed segmentation software *FireVoxel* [11]. Segmentation was carried out by an experienced operator together with a musculoskeletal radiologist in a consensus session. The femur was manually outlined using an adjustable paintbrush/eraser tool controlled by a computer mouse, with the ability to zoom into image subregions and switch interactively between painting and erasing modes. Additional filling and morphing tools were used to accelerate the process. The segmentation employed *FireVoxel*’s MagTrace and edge parameters (multiscale texture gradient). The following parameters were used: snap‐to‐edge radius = 10 voxels; spline tension = 0.5; edge parameters, low radius = 6.070 voxels; distance metric = Earth mover distance; voxel weights = constants; magnitude measure = normalized; normalization coefficient = 1.

### 2.7. Image Processing

MR images were segmented using FireVoxel software by delineating the periosteal border of the whole proximal femur and the acetabulum [12] as described above. To account for partial volume effects between the bone and marrow voxels, grayscale values of the image were linearly scaled between 0 (for pure marrow) and 100 (for pure bone) using local thresholding [13]. A bone volume fraction (BVF) map was then generated, consisting of a three‐dimensional array representing the fractional occupancy of bone at each voxel location.

### 2.8. Finite‐Element‐Derived Bone Strength

Patient‐specific proximal femur strength was obtained via finite‐element analysis using a solver specifically designed for modeling microstructural bone images [14]. A microlevel finite‐element model was constructed using the derived BVF maps. Each voxel in the BVF map was modeled with dimensions equal to the three‐dimensional voxel resolution and the tissue modulus of elasticity, which was proportional to the grayscale intensity range [6]. 100% intensity was assigned a value of 15 GPa for bone tissue, and Poisson’s ratio was set to 0.3. Similar to previous studies, sideways fall behavior was mimicked by mechanical simulations of the force exerted by the contact region of the femoral head and the greater trochanter where the diaphysis was angled at 10° with respect to the ground with 15° of internal rotation [10]. Nonlinear behavior of tissue elastic modulus was simulated via a hyperbolic secant function at each element [6]. The displacement of the femoral head was measured while the greater trochanter on the opposite surface was constrained. As the displacements increased, the reaction force of the femoral head increased until the point of fracture, after which the force decreased. Each model was run until a predetermined strain of 3% strain. The bone strength could be calculated from the maximum point of the resulting nonlinear force–displacement curve. This approach has repeatedly been shown to offer strong correlations with the failure load derived from the mechanical testing of cadaveric human femora [8].

### 2.9. Measuring STT

**Figure 1:**
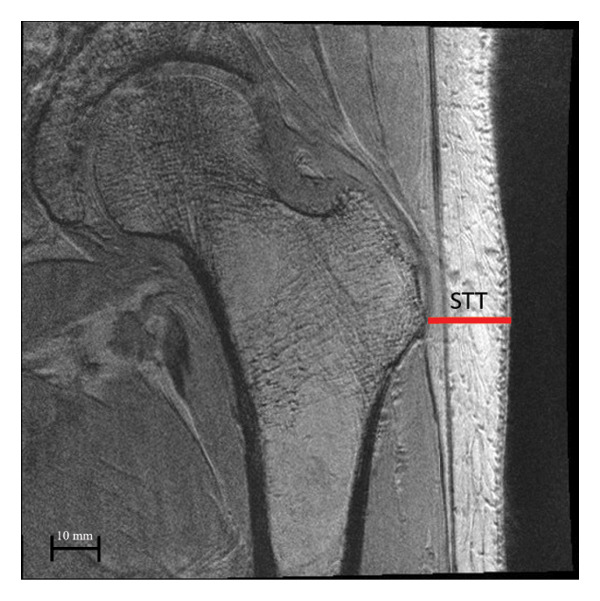
Derivation of trochanteric soft tissue thickness (STT) using patient MR images. STT was calculated as the distance between the greater trochanter and the skin.

Using the MR images, the trochanteric STT was calculated from a coronal MR slice, showing the least distance between the greater trochanter and the skin as shown in Figure 1.

### 2.10. Metrics for Comparison

In the comparison between hip fracture and matched controls, DXA‐derived T‐scores, Z‐scores, and BMD were calculated at the time of recruitment. Values were derived from the patient’s prior medical records that were closest to the MRI date. FRAX scores including the major osteoporotic fracture, major osteoporotic fracture (without BMD), hip fracture, and hip fracture (without BMD) were also reported and compared across the two groups. DXA was done within 6 months of MRI.

### 2.11. Statistics

The Anderson–Darling test was used to assess normal distribution for all factors. The mean ± standard deviation or the median and interquartile range were reported in​ Table 2 for normal and non‐normal distributions, respectively. For normal distributions, a one‐way two‐sample *t* test comparing the metric between hip fracture (*n* = 10 subjects) and nonhip fracture patients (*n* = 10 subjects) was conducted using JMP software (Table 2). For non‐normal distributions, significance was assessed through a nonparametric Wilcoxon rank sum test. For all tests, alpha was set at 0.05. These tests were used to identify parameters that displayed significant differences between fractured and nonfractured groups to validate our models and assess their predictive nature.

**Table 2:** Comparisons of patient characteristics and other standardized scores between hip fracture and nonhip fracture patients.

Measure	No hip fracture	Hip fracture	*p*
Bone strength index (BSI)	0.778 [0.570–1.05]	0.548 [0.301–0.741]	**0.0455**
FEA failure load (kN)	3.60 [3.13–4.95]	2.83 [1.41–3.95]	0.131
Metric‐based BSI			
Age‐based BSI	1.04 [0.62–1.86]	5.46 [−7.24–18.2]	0.286
Weight‐based BSI	1.10 [0.68–1.52]	5.85[−7.45–21.9]	0.270
Height‐based BSI	1.25 [0.72–1.78]	7.13 [−9.95–23.8]	0.280
STT‐based BSI	1.30 [0.53–2.07]	15.8 [−12.2–43.8]	0.280
Impact force (kN)			
Age‐based	3.46 [3.15–4.12]	3.50 [3.15–4.15]	0.909
Weight‐based	3.98 [3.87–4.14]	4.06 [3.93–4.26]	0.273
Height‐based	4.55 [4.21–4.89]	4.73 [4.45–5.10]	0.363
STT‐based	4.42 ± 0.692	5.340 ± 0.560	0.0699
Maximum	4.89 [4.55–5.18]	5.13 [4.50–5.79]	0.520
Mean	4.03 [3.91–4.42]	4.35 [3.82–4.73]	0.650
Patient characteristics			
Age (yrs)	64.0 [54.0–68.8]	63.5 [53.5–68.8]	0.909
Weight (kg)	52.4 [47.0–60.1]	56.2 [50.2–65.8]	0.273
Height (m)	1.60 [1.54–1.65]	1.63 [1.58–1.68]	0.363
STT (mm)	34.3 ± 8.78	22.6 ± 7.10	0.0699
DXA			
T‐score			
Femoral neck	−2.1 [−2.9–−1.6]	−1.6 [−2.6–−1.1]	0.246
Total hip	−2.0 [−2.8–−1.5]	−2.1 [−2.5–−0.85]	0.658
Lumbar spine	−2.6[−3.1–−1.5]	−1.3 [−2.2–−0.5]	0.0949
Z‐score			
Femoral neck	−1.4 [−1.5–−1.3]	−1.20 [−1.6–−0.80]	1.00
Total hip	−1.6 [−1.8–1.3]	−1.80 [−2.1–−1.5]	0.439
Lumbar spine	−1.3 [−1.5–−1.1]	−0.450 [−1.9–1.0]	1.00
BMD (g/cc)			
Femoral neck	0.760 [0.684–0.835]	0.667 [ 0.528–0.749]	0.248
Lumbar spine	0.958 ± 0.0764	0.952 ± 0.247	0.978
FRAX			
Major osteoporotic fracture	11.0 [7.8–23.0]	20.0 [16.8–30.5]	0.147
Major osteoporotic fracture (without BMD)	17.0 [9.88–23.0]	20.5 [11.5–32.5]	0.400
Hip fracture	2.4 [1.4–6.0]	3.90 [2.5–7.25]	0.324
Hip fracture (without BMD)	4.10 [1.65–8.48]	5.55 [2.73–7.00]	0.834

*Note:* Bolded *p* values indicate the significance.

## 3. Results

### 3.1. Computational Model Development

The effects of age (yrs), weight (kg), height (m), and STT (mm), on impact force (kN) collected from the prior literature are summarized in Table 3, which describes the number of sample points from each study and a general description of the demographics and techniques used to compare the variable of choice against the impact force. The models derived from these sources were estimated as

(1) FkN=−0.10310.2∗age+,FkN=0.007560.00555∗weight+,F kN=4.552.72∗height−,FkN=−0.07237.10∗soft tissue thickness+.

**Table 3:** Literature sources pertaining to each factor and derived relationships.

Factor (x)	Reference	Number of samples	Study description	Technique for impact force
Age (yrs)	Lots and Hayes, [15]	12	Proximal part of the femora from cadavers with age range 53–81 years (69 ± 9 years) at death	Finite‐element analysis and quantitative computed tomography as an indirect measure of bone density
	Courtney et al. [16]	14	Seven female and seven male volunteers aged 20–35 years	Pelvis‐release experiments—low force, zero‐initial velocity falls on the hip combined with analytically derived parameter models

Weight (N)	Majumder et al., [17]	7	Adult male subjects from 63 to 93.4 kg	Finite‐element analysis models.
	Luo et al.,[18]	3	Young male volunteers from 64.6 to 75.6 kg	Subject‐specific dynamic models derived from DXA images and controlled fall tests

Height (m)	Majumder et al., 2013 [17]	7	Adult male subjects from 63 to 93.4 kg	Finite‐element analysis models
	Majumder et al., [19]	3	Young male volunteers from 64.6 to 75.6 kg	Subject‐specific dynamic models derived from DXA images and controlled fall tests

Soft Tissue Thickness (mm)	Majumder et al., [19]	98	Slices of tissue complex from 58‐year‐old male	Finite‐element simulations of sideways fall
	Majumder et al., [17]	7	Adult male subjects from 63 to 93.4 kg	Finite‐element analysis models.
	Robinovitch et al., 1995 [20]	9	Samples of soft tissue form the trochanteric regions of nine cadavers from three men and six women with age range 60–102 years (77 ± 10 years) and body weight 294–942 N (559 ± 245 N)	Mechanical testing experiments

**Figure 2:**
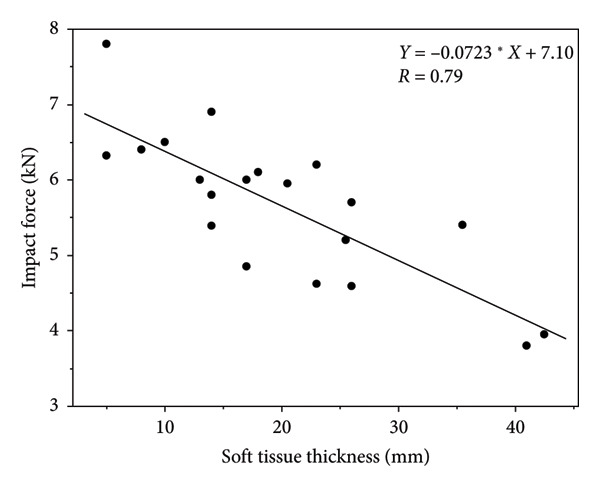
Literature‐derived model relating soft tissue thickness (mm) and impact force (kN) during a fall on the hip.

**Figure 3:**
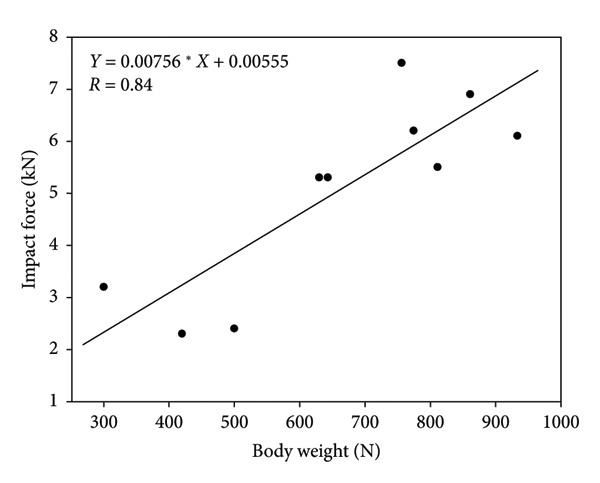
Literature‐derived model relating body weight (N) and impact force (kN) during a fall on the hip.

**Figure 4:**
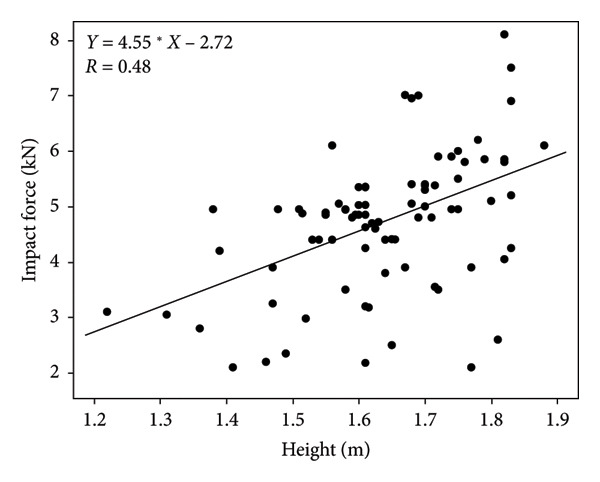
Literature‐derived model relating height (m) and impact force (kN) during a fall on the hip.

**Figure 5:**
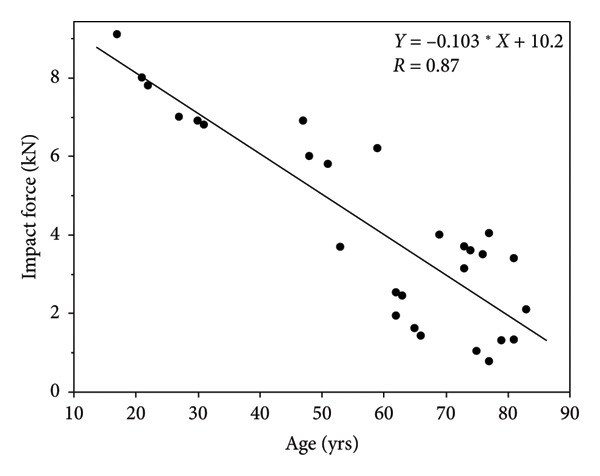
Literature‐derived model relating age (yrs) and impact force (kN) during a fall on the hip.

A graphical illustration of the derived model in relation to the literature sources they were derived from can be found in Figures 2, 3, 4, 5. Even after statistically adjusting for height and weight, age was found to be an independent predictor of maximum impact force (*p* < 0.0001). The maximum impact force was calculated from the maximum value among the four models. The distribution of factors that equated to the maximum impact force between the controls and treatment group is summarized in Table 4. 55% (11/20) of the maximum impact forces were derived from the height metric, 25% (5/20) of the maximum impact forces were derived from STT, and 10% (2/20) of the maximum impact forces each were derived from age and weight. This suggests that height as a distinct factor may have the strongest correlation with impact load.

**Table 4:** Distribution of factors that yielded the maximum impact force.

Factor	Control (*N* = 10)	Hip fracture (*N* = 10)
Age (yrs)	1	1
Height (m)	7	4
Weight (kg)	0	2
Soft tissue thickness (mm)	2	3

### 3.2. BSI Derivation and Validity Testing

The BSI was derived by dividing the bone strength by the maximum impact force calculated across the four metrics. The index was validated by comparing 10 patients without hip fractures to 10 patients with prevalent fractures matched for age, race, and BMI from the ages of 50–90 years. There were no individual differences in patient‐derived characteristics between the two groups (Table 1). The total BSI was found to be lower in hip fracture patients (*p* = 0.0455) (Table 2). There were no significant differences, however, in FEA failure load, DXA‐derived scores, or any of the FRAX score parameters between the two groups. There was also no difference in the FEA‐derived bone strength and impact force when individually compared between the two groups, highlighting the collaboration of both factors in the overall index in determining overall fracture risk. This was further seen as those with the highest bone strength did not necessarily correlate with the lowest impact force. The mean BSI in the hip fracture group was 0.536, and no hip fracture group was 0.862. The mean difference in the BSI (Yes − No) was −0.326 with 95% CI (−0.642, −0.009) and Cohen’s d−0.97.

The maximum impact force utilized in the BSI was calculated from the maximum value among the four models. However, when BSI was calculated based on a singular metric rather than the maximum of them all, there was again no difference between the two groups suggestive of the importance of an aggregate comparison of maximum impact force derived from various factors. The data used for generating the regression models could be made available for researchers who request the data for academic purposes.

## 4. Discussion

In the present study, we were able to accomplish two aims: (1) We developed predictive models for impact force based on age, weight, height, and STT. (2) We introduced the new measure to predict future risk called the BSI, which denotes the ratio of a patient’s bone strength to their maximum impact force derived from the regression models. We then validated this approach by comparing the results of the regression model with a retrospective sample of patients with or without known hip fractures.

There was a notable decrease in the total BSI among hip fracture patients compared to nonfractured patients, indicating the usefulness of combining structural bone integrity measures with other patient‐specific factors. Among those with diagnosed osteoporosis, there was a similar decrease in the BSI among hip fracture patients compared to control patients (*p* = 0.0392). The sample was not large enough to draw conclusions about the patients with osteopenia in the study.

Individual analysis of the components that comprise the BSI calculation (i.e., impact force and FEA) showed no significant difference. The similarity in FEA failure load coincides with the recent research, suggesting the improved yet limited utility of FEA in fracture assessment. The lack of difference in any of the individual patient metrics suggests that a regression model based on these factors uniquely may not hold predictive power for future hip fractures. They may, however, act in concert or coincide with other factors as suggested by the significant decrease in the BSI in hip fracture patients. The maximum impact force derived from STT measurements did not reach significance and however demonstrated a trend suggestive of decreasing STT contributing to a decrease in the BSI (*p* = 0.0699). The diversity in metrics that resulted in the maximum impact force further supports the cumulative use of a variety of patient characteristics for BSI calculations. It should further be noted that while STT measurements in the study were derived from MRI, they can also be calculated from other imaging techniques such as CT, x‐ray, or DXA.

The DXA and FRAX scores proved to be an otherwise unreliable predictor of future hip fractures. It would be expected that among patients with a high risk of fracture, the DXA score should be considerably less in individuals predicted to sustain an osteoporotic fracture. Similarly, FRAX scores should be significantly greater. However, in the present study, there was no difference in these scores between the fracture and control groups. These findings coincide with recent literature that suggests DXA and FRAX scores should not be used as the sole indicator for osteoporotic fracture risk [21].

Our study has a number of limitations. First, the study is limited by the number of samples. In order to derive a more comprehensive, representative model, more samples from additional literature should be incorporated in the overall model. A larger sample size for comparison between hip fracture and nonhip fracture patients in testing the utility of our computational approach should also be considered. A larger sample size may better reflect the significant differences seen in various metrics and fracture scores between hip fracture and controls. For example, our data showed that the failure load was ∼25% less in hip fracture patients compared to controls. Despite this large difference, the difference did not reach statistical significance (*p* = 0.131), potentially due to the small sample size. Furthermore, we did not perform Bonferroni correction to control for multiple comparisons because our study was exploratory in nature requiring larger validation studies.

The literature used in our study range from a wide set of dates and methodologies. Future studies should consider focusing the search on specific date ranges and testing methods. An additional limitation was that univariate models were derived without individual adjustment of covariates that may have arisen when comparing patients across different studies. In the future, more care toward grouping patients based on specific metrics, while keeping other variables as consistent as possible, should be attempted to develop concise models. Alternatively, future studies could investigate the utility of multivariate predictors of maximum impact force. Lastly, models were derived by averaging the slope and intercept of the linear equations obtained from the literature. Averages did not take into the account different sample sizes between studies. In future iterations, it may be worthwhile to consider taking weighted averages of these parameters to adjust for differences in sample sizes. Aside from optimizing literature collection and the subsequent synthesis of data, future work could explore further validation of impact force estimations. In terms of the patient population used, the fracture group comprised individuals with previous fractures from before their MRI scans. In order to more accurately assess the ability of predicting future fractures, further studies will need to apply these methods to a group with incident fractures.

Overall, the analysis of patient data from both prior literature studies and sample studies shows the potential to improve fracture risk in osteoporosis patients through a combination of simple, quantitative regression models and finite‐element analysis. While singular models relating a patient metric such as age, height, weight, or STT to predicted impact force during a fall were not different between osteoporotic patients with fractures compared to without fractures, this study suggests that utilizing the results of these models in conjunction with one another may prove to be a reliable patient‐specific FRAX. Calculating bone strength using finite‐element analysis incorporates the effects of the bone architecture in addition to bone mineral density, which is the only measurement used in standard fracture risk calculators. Comparing the maximum impact force from patient‐specific regression models with the finite‐element analysis–derived bone strength in the present study allowed for the calculation of the BSI, which was found to be dramatically reduced in patients with hip fracture. There was no difference in the DXA or FRAX scores between the two groups coinciding with recent literature suggesting their inefficacy in accurately predicting fracture risk, despite their wide adoptability. Future literature research and analysis will need to examine more concise models reflective of various differences such as gender and potentially to identify trends between other patient‐specific measures and their association with impact force during a fall. The optimization of these models could pave the way for a more comprehensive and patient‐specific hip fracture prediction that better accounts for the variable factors contributing to bone health.

## 5. Conclusion

The implementation of easy to use, quantitative regression models to assess future hip fracture risk can have extensive benefits in determining preventative care given to patients with osteoporosis and other bone‐related conditions. Literature sources have correlated a variety of metrics from patient data with predicted proximal femur impact force during a sideways fall. We can combine these findings into aggregated regression models designed to predict future fracture risk. The results found in the present study preliminarily suggest that the combination of various patient metrics with finite‐element analysis–derived bone strength may supplement DXA‐derived and FRAX scores used in the current standard of care to provide a comprehensive osteoporotic hip fracture assessment. Further studies, however, with larger sample sizes and more precise methods of model derivation will be needed to optimize accuracy.

## Ethics Statement

The University of Pennsylvania Institutional Review Board approved this study.

## Consent

Informed consent was obtained from all individual participants included in the study.

## Conflicts of Interest

The authors declare no conflicts of interest.

## Author Contributions

Study design: Chamith S. Rajapakse and Gregory Chang. Literature review: Alisha Agarwal, Nishtha Gupta, and Chamith S. Rajapakse. Data analysis: Alisha Agarwal, Nishtha Gupta, Daniel Kargilis, and Chamith S. Rajapakse. Manuscript writing and editing: Alisha Agarwal, Nishtha Gupta, Daniel Kargilis, Gregory Chang, and Chamith S. Rajapakse.

## Funding

The authors would like to acknowledge the grant support from [NIH R01 AR068382] and [R01 AR076392].

## Data Availability

The data that support the findings of this study are available from the corresponding author upon reasonable request.
